# Childhood Cognitive Ability Predicts Adult Financial Well-Being

**DOI:** 10.3390/jintelligence5010003

**Published:** 2016-12-27

**Authors:** Adrian Furnham, Helen Cheng

**Affiliations:** 1 Department of Psychology, University College London, London WC1E 6BT, UK; h.cheng@ucl.ac.uk; 2 BI: Norwegian Business School, Nydalsveien 37, 0484 Oslo, Norway; 3 ESRC Centre for Learning and Life Chances in Knowledge Economies and Societies, Institute of Education, University College London, London WC1H 0AL, UK

**Keywords:** earning ability, childhood cognitive ability, educational qualifications, occupational prestige, personality traits, longitudinal

## Abstract

This study set out to investigate to what extent childhood cognitive ability, along with personality traits, education and occupational status, as well as marital status influence adult financial success. Data were drawn from a large, prospective birth cohort in the UK, the National Child Development Study (NCDS). The analytic sample was comprised of 4537 cohort members with data on parental social class (at birth), cognitive ability (at age 11), educational qualifications (at age 33), personality traits (at age 50), current marital status and occupational prestige, and salary/wage earning level (all measured at age 54). Correlational results showed that parental social class, childhood cognitive ability, traits extraversion, emotional stability, conscientiousness, and openness, being married positively, being divorced or separated negatively, education and occupation as well as gender were all significantly associated with adult earning ability (*p* < 0.05 to *p* < 0.001). Effect sizes for the relationship between intelligence and income was moderate. Results of a multiple regression analysis showed that childhood cognitive ability, traits conscientiousness and openness, educational qualifications and occupational prestige were significant and independent predictors of adult earning ability accounting for 30% of the total variance. There was also a gender effect on the outcome variable. Numerous limitations are noted.

## 1. Introduction

Differential psychologists have long been interested in correlates and predictors of work success.

Over the years, both personality/preference and intelligence/ability psychologists have attempted to determine which, why and how individual difference factors predict various measures of success at work. As a consequence, there are various reviews of the literature, specifically with regard to personality [1] and intelligence [2]. They have attempted to understand the mechanisms and processes that account for the relationship.

There have been many papers on correlates of work success and general life outcomes [3]. For instance, Roberts et al. [4] in a meta-analysis of hundreds of longitudinal studies showed intelligence, personality and socio-economic status had similar significant effects on occupational attainment as well as divorce and mortality.

Indeed, there have been so many studies that various meta-analyses have been published [5,6]. Ng and Feldman [6] reviewed 216 samples and found clear evidence of personality correlates of subjective work success with low scores on four traits (emotional stability, extraversion, openness, conscientiousness) and high scores on one trait (agreeableness) being associated with less success. Higher educational level and cognitive ability were also related to success in the review.

The issue is that mean-level differences across professional groups/jobs on either traits or salary may obscure within-group relations between these variables. For instance, trait agreeableness may be negatively associated with salary because agreeable individuals tend to choose lower-paid professions; however, within-occupations agreeableness is positively associated with income. This is because agreeable individuals tend to be better leaders and coworkers, enabling them to build better social networks and obtain more resources/rewards from others. That is, traits affect both choice of, and performance in, a job, both of which are related to performance.

The influence of traits on career success between-groups versus within-groups are, however, different questions. Between-groups analyses, such as used in this study, mix the influence of traits on occupational choice/entry and performance as contributors to income. Within-groups/single-occupation samples isolate the effect of traits on performance. Neither design is necessarily better as they can answer different questions.

The current paper looks at childhood intelligence as one predictor of adult salary. It is a longitudinal, between occupational group study that considers other potentially relevant variables in this relationship. There are three important issues in this area.

### 1.1. The Definition of Work Success

Work or career success has been defined as the accumulated positive work and psychological outcomes resulting from one’s work experiences [7]. Success has usually been operationalized as intrinsic and extrinsic criteria: extrinsic indicators of success are such things as physical and material rewards received from one’s employer such as salary and job title [8]. Intrinsic measures include feelings or ratings of satisfaction or contentment with career and psychological rewards received. These attempt to capture an individual’s subjective judgments about their career achievements [9]. Most studies in this area rely on self-reports for both extrinsic and intrinsic motivation, which is not problematic as individuals report their income fairly accurately when there are no consequences for doing so, as in this study [10]. Furnham [1] has also noted that, as may be expected, extrinsic and intrinsic success are moderately related.

Many studies in this area have used a mix of measures. Sutin, Costa, Miech and Eaton [11] used occupational prestige (job title), annual income and self-reported job satisfaction. Some studies using both extrinsic (salary) and intrinsic (career satisfaction) measures found they had different antecedents like management style and opportunities for advancement [12].

As noted above, there are a whole range of criteria that may be considered to be markers of occupational success: salary, rank, job title, speed of promotion, etc. There are essentially three problems associated with these variables. The first is that it is problematic to compare across jobs, organisations or sectors. Second, there are great differences between regions, countries and time periods. Third, in times of growth (which may be specific to a sector, or region or product), people are likely to be paid more than in times of stagnation. Despite limitations of this measure in this study, we used subjectively reported current net payment per month as the criterion variable.

### 1.2. Multiple Variables

Differential psychologists tend to concentrate on their own variables as predictors of work outcomes.

Many of the social sciences (economics, psychology, sociology) as well as financial planners and policy makers are interested in the predictors and correlates of adult financial well-being and earnings. Economists look at structural factors, while sociologists tend to focus on parental social class and education [13,14].

Economists have shown that education level, schools and universities attended and type of degree are strong predictors of income. Education shows people how to access and critique information and increase verbal, numerical and financial literacy. Sociologists have shown that parental social class is a strong predictor of job success. Families pass on not only genes but social environments. They socialize values like prudence, thrift, and postponement of gratification, all of which are important in career success. People of higher social class provide more stable and enriched environments where learning and achievement are encouraged. Social class is related to wealth and early access to all sorts of things that influence young people.

There are various studies that have looked at the simple idea that you may “have to be smart to be rich” (p. 489) [15]. Equally, personality psychologists have tried to stipulate which traits (i.e., conscientiousness) predict career success [11,16]. However, the majority of studies have examined more than one set of individual differences often combining intelligence and personality [17,18].

Recently, Wai and Lincoln [19] looked at over 18,000 high net worth individuals. The results were as predicted: “cognitively able” people tended to be better educated and therefore had better jobs with higher salaries. The authors noted the “clustering of brains, wealth and power” which lead to elite education, which, in turn, helped the “trajectory” of money making.

In this study, we examine gender, marital status, educational attainment, occupational prestige, and personality as well as intelligence as predictors of salary.

### 1.3. Cause and Consequence

A great deal of psychological research is cross-sectional rather than longitudinal, though there are meta-analyses of longitudinal studies [2,4]. Sutin et al. [11] reported on 731 American adults and used job title, annual income and job satisfaction as outcome measures. As has been found before, the emotionally stable and conscientious people were both richer (higher salary) and happier (greater job satisfaction). They also found in the longitudinal part of the study that extraversion predicted income over a 10-year period.

In a study on over 7000 American baby-boomers, Zagorsky [15] found an increase in one IQ point was related to an increase of $234 and $616 per annum. Taking into account other potentially confounding variables, Abele and Spurk [20] examined how objective/extrinsic and subjective/intrinsic measures of career success interrelate over time. They found that measures of subjective success had a strong influence on the growth of objective success. They concluded that subjective ratings influenced objective ratings more than vice versa, suggesting the power of self-confidence in career success. This suggests that, as in all research in this area, it is best to have multiple measures of both objective and subjective measures if one really wants to understand the problem.

Spurk and Abele [21] looked at 432 Germans over a four-year period measuring them three times. Their theory was that personality determines two motivational factors: a sense of occupational self-efficacy and career advancement goals that determines their contractual hours, which, in turn, predicts salary.

Le, Donnellan and Conger [22] tested the relationship between personality, specifically positive and negative emotionality and income. They found, as predicted, that agentic positive emotional stability was a consistent and positive predictor of income over time.

In a study of nearly 5000 Americans, Converse, Piccone and Todd [23] postulated and demonstrated that self-control in childhood was a good predictor of education, which predicted job complexity that was related to salary.

#### 1.3.1. The Current Study

In this study, we examine the role of intelligence measured at 10 years old, parental social class at birth, education at 33 years old, personality at 50 years old and occupational prestige and income at 54 years old. This study aims to investigate gender differences, psychological (childhood intelligence and adult personality) and sociological (parental social status, educational achievement, occupational prestige) factors in a longitudinal sample to investigate how they together related to adult earnings defined by net monthly income. Some of these variables are inevitably highly correlated like occupational prestige and income with typical correlations around *r* = 0.5 [1]. The focus is particularly on the individual difference variables (intelligence and personality) and to what extent they predict adult earnings.

There are established findings on the links between family background, early cognitive development and later educational and occupational outcomes [24,25,26,27]. In general, intelligent people and those who came from higher social backgrounds are more likely to have higher educational qualifications and attain higher occupational levels and therefore have higher earnings. Hence, one would expect a direct and an indirect link from childhood intelligence to adult earnings.

Some studies in the area are longitudinal, and there has been an established causal direction of these variables: education predicts occupation, intelligence predicts educational outcome, intelligence is associated with career success, and occupational prestige is associated with earnings [24,28,29,30,31,32,33,34]. Few studies have looked at the effects of family background, childhood cognitive ability test scores, and later educational and occupational outcomes on adult earning ability in relation to personality factors. Moreover, as Geyer et al. [31] noted, education, income and occupational class cannot be used interchangeably in social epidemiology. Many studies in the area used occupation and income as indicators of a latent variable. This study will overcome the above-mentioned by using these measures separately.

Whilst there are a few early studies on personality traits and earning [35], it was Bowles, Gintis and Osborne [36] who concluded that non-cognitive traits are important in determining earnings that stimulated this field most. Early studies showed cultural differences in the relationship between personality and remuneration: neuroticism was associated with lower levels of extrinsic success for American executives but not the Europeans, while extroversion was associated with higher levels of extrinsic success for the European executives but not the U.S. executives [37]. However, the studies have been inconsistent on which personality traits are measured [18,21,38,39]. Linz and Semykina [40] concluded “the effect of personality is similar in magnitude to the effect of education, and may, in fact, exceed the effect of education if the effect of two personality traits is combined” (p. 71).

Psychological studies of work success (in general) using the established “Big Five” traits have concentrated on personality factors [41], physical characteristics [42,43], demographic variables like age, class and gender [44] as well as intelligence [45]. Nearly all the studies have shown that two personality traits, namely neuroticism (poor emotional adjustment) and conscientiousness, are, by far, the most important in explaining the variance of success at work measured by promotion, ratings, level, and also pay [11]. Whilst some studies have looked at the reciprocal effect of work experience on personality development [4], most have looked at personality predictors of job choice and success. Others have suggested that, while personality may be a direct predictor of salary, it is mediated by motivation and work status [21].

Using a Dutch household survey database of 828 workers, Nyhus and Pons [46] found that emotional stability was positively associated with pay in both men and women and that the “economic returns” of personality factors in pay varies between educational groups and gender. Using a larger number of the same dataset, Gelissen and de Graaf [47] found extraversion was positively and openness negatively related to earnings but only for men. They concluded “that personality characteristics contribute importantly to earnings and status attainment, in addition to sociological variables” (p. 702).

The psychological studies examining ability and personality predictors of work success and adult earnings have typically three limitations: many are cross-sectional rather than longitudinal so that causality cannot be inferred; the samples tend to be small and non-representative of the total population; they tend to be very restricted in the variables measured in that they may measure personality, but not also intelligence, or education, which may be powerful moderating or mediating factors. Furthermore, some do not look at the role of education, or, indeed, occupational attainment, which are very obvious correlates of earnings and may be moderator or mediator variables. This study hopes to overcome these shortcomings.

There have been numerous studies on the possible causes of the established gender difference in pay, which include gender differences in education, hours worked, occupational prestige, employment sector and years in the labour market [48,49].

Semykina and Linz [50] found gender differences in personality traits that explained 8% of the gender wage gap. They also found women’s earnings are strongly affected by personality, while the effect of personality on men’s earnings was small and often not significant. This study considers the effects of cognitive and non-cognitive individual difference factors separately for males and females.

#### 1.3.2. Hypotheses

There were two research questions of interest in the present study: first, we aimed to ascertain whether childhood cognitive ability has long lasting effects on adult earning ability, and, second, how personality factors influence earning ability. We sought to look at the effects of intelligence and personality as direct as well as indirect correlates of financial status. Based on the literature reviewed above, it was hypothesised that: (1) childhood cognitive ability would have a significant effect on adult earning ability; (2) openness would be significantly associated with the outcome variable; and (3) being married would be significantly associated with the outcome variable.

## 2. Method

### 2.1. Participants

The National Child Development Study 1958 is a large-scale longitudinal study of the 17,415 individuals who were born in Great Britain in a week in March 1958 [51]. There were nine follow-ups. The latest data were collected when cohort members were 54 years old. At 54 years old, 8958 participants were interviewed (response = 78%) and 4984 participants provided information on their current net payment (response = 56%). The analytic sample comprises 4537 cohort members (53 percent females) for whom complete data were collected at birth, at age 11, at age 33, at age 50, and at age 54. Bias due to attrition of the sample during childhood is potentially a problem [52,53]. Participants could elect to decline disclosure to any question and it cannot be certain why some did or did not report their salary and whether there were any systematic differences between them.

### 2.2. Measures

1. Parental Social Class. Parental social class at birth was measured by the Registrar General’s measure of social class (RGSC). RGSC is defined according to occupational status [54]. Where the father was absent, the social class (RGSC) of the mother’s father was used. RGSC was coded on a six-point scale (numbers in brackets represent percentages in this study: I professional (6.1%); II managerial/technical (40.5%); IIIN skilled non-manual (22.0%); IIIM skilled manual (17.0%); IV semi-skilled (12.3%); and V unskilled (2.1%) occupations [55].
2. Childhood Cognitive Ability. Childhood cognitive ability was assessed at age 11 in school using a general ability test [56] consisting of 40 verbal and 40 non-verbal items. For the verbal items, children were presented with an example set of four words that were linked either logically, semantically, or phonologically. For the non-verbal tasks, shapes or symbols were used. The children were then given another set of three words or shapes or symbols with a blank. Participants were required to select the missing item from a list of five alternatives. Scores from these two set of tests correlate strongly with scores on an IQ-type test used for secondary school selection (*r* = 0.93, [56]), suggesting a high degree of validity.
3. Educational Qualifications. At age 33, participants were asked about their highest academic or vocational qualifications. Responses are coded to the six-point scale of National Vocational Qualifications levels (NVQ) ranging from ‘none’ to ‘higher degree level’: 0 = no qualifications; 1 = some qualifications (Certificate of Secondary Education Grades 2 to 5); 2 = O level (equivalent to qualifications taken at the end of compulsory schooling); 3 = A level (equivalent to university entrance level qualifications); 4 = diploma and equivalent; and 5 = university degrees and equivalent.
4. Personality Traits. Personality traits were assessed at age 50, by the 50 questions from the International Personality Item Pool (IPIP) [57]. Responses (5-point, from “Strongly Agree” to “Strongly Disagree”) are summed to provide scores on the so called ‘Big-5’ personality traits: extraversion, emotional stability/neuroticism, conscientiousness, agreeableness and intellect/openness. Scores on each trait range between 5 and 50 with higher scores equating to higher levels of each trait, of which 10 items for extraversion, 10 items for emotional stability, 10 items for agreeableness, 10 item for conscientiousness, and 10 items for intellect. Alpha was 0.73 for extraversion, 0.88 for emotional stability, 0.81 for agreeableness, 0.77 for conscientiousness, and 0.79 for openness.
5. Marital Status. At age 54, cohort members provided information on their current marital status. The derived measure contains four groups: “married” (73%), “never married” (9.3%), “divorced/separated” (15.7%), and “widowed” (2%). Because of the small number in the group “widowed” (*n* = 91), this group was excluded from further analyses. Dummy variables were created for the following analyses. Married (1 = married, 0 = not married); never married (1 = never married, 0 = not never married); divorced/separated. (1 = divorced/separated, 0 = not divorced/separated).
6. Occupational Prestige. Data on current or last occupation held by cohort members at age 54 are coded according to the RGSC described above, using a 6-point classification.
7. Adult Earning Ability. At age 54, participants were asked about their current net payment per month. A small proportion of observations with very low or very high incomes are dropped (81 cases) from the sample so that the results are not unduly influenced by these outliers, and incomes were logged in the following analyses.

## 3. Results

### 3.1. Correlational Analysis

Correlation analysis was conducted to examine the associations between adult earning ability and a set of psychological and socio-demographic variables in the study (see Table 1).

Inevitably, with the sample size in this study, virtually all of the correlations were significant even though trivial in size. Correlations *r* > 0.30, could be considered large. Table 1 shows that among the variables examined in the study education and occupation were significantly associated with adult earning ability (*p* < 0.05 to *p* < 0.001). However gender, cognitive ability and trait openness could be considered to have a moderate effect (*r* > 0.20) on income.

Table 1 also shows that childhood cognitive ability was significantly associated with education and occupational prestige, and to a lesser extent, trait openness. Further, being married and divorced/separated were significantly and inversely associated with the outcome variable. Thus all three hypotheses were supported.

### 3.2. Regression Analysis

Following this, a multiple regression was carried out. Table 2 shows the results.

Table 2 shows that childhood cognitive ability, traits conscientiousness and openness, educational qualifications and occupational prestige were significant and independent predictors of adult earning ability accounting for 30% of the total variance. Based on meta-analyses of thousands of studies in applied psychology, Paterson et al. [58] found that correlations of 0.10, 0.20, and 0.30 corresponded to small, medium, and large correlations. These values are the 25th, 50th, and 75th percentiles for all correlations observed in applied psychology.

Clearly, the results demonstrate that occupation, education and gender are the most powerful predictors of salary, which is well established. However, the results also implicate cognitive ability, conscientiousness and openness as playing a small, but explicable, role. It appears that much of intelligence’s influence on adult earnings results from indirect effects by contributing to higher levels of education and occupational prestige.

We conducted other analyses such as step-wise regressions and separate regressions for males and females, though they did not change the following conclusions.

## 4. Discussion

This study examined two main components of individual differences (intelligence and personality) in influencing adult earning ability together with socio-demographic variables. The results showed that childhood cognitive ability has a modest but long lasting effect on adult earning ability 43 years later. It also showed that personality traits measured four years earlier were correlated with the outcome variable.

The correlation between intelligence, measured robustly at age 10, and their monthly income 43 years later was *r* = 0.24. This is a modest but important relationship. This result is not surprising and replicates other studies. The mechanism explaining this association is also well established: brighter people tend to have higher (and better) educational qualifications, which, in turn, lead them to obtaining better paid jobs. Indeed, the highest correlations in Table 1 (all *r* > 0.45) were between educational level and occupational prestige, intelligence and educational level as well as income and occupational prestige. What is particularly interesting is how young the participants were when their IQ was measured and its effects many years later.

There is also the interesting and important question of change in cognitive ability and personality over time. There is an extensive literature on whether personality changes much over time with some people adopting the “plaster” or little change, as opposed to the “plastic” or greater change perspective [59]. There has been less work on the stability of intelligence (IQ) over time, though there is still debate between those with different *mindsets* as defined by Dweck [60]. However, the extant literature implies that IQ is surprisingly stable over time suggesting that early (around adolescent) measure, but reliable measurement, is highly predictive of many life outcomes [61].

The results of this study showed that personality traits were also correlated with income, though the regressions suggested that two were the most important. This study found, as many others have, that conscientiousness was positively correlated with income [62]. Conscientious people tend to do better at work and rise to higher positions which are better paid. This study also showed openness to be positively associated with income, particularly in the correlational result (*r* = 0.22). This result may be explained by the well-established correlation between openness and intelligence (*r* = 0.25). Open people tend to be more curious, to have more positive attitudes to learning and experimentation, and hence better educational outcomes (*r* = 0.31), which may promote success at work and better jobs (*r* = 0.26).

The results showed two factors *not* directly related to adult monthly income. The first was parental social class. The correlational results showed, as expected, that parental social class was correlated with the participants’ intelligence, education and occupational status, as has been shown many times before. However, social class was not significant in the regression and the results could be interpreted to suggest that the main effect of parental social class is to facilitate education, which directly impacts occupational status.

The results did, however, show a very interesting and significant effect for gender. The results showed most interestingly that whilst the females were brighter than the males (at age 11), they achieved lower educational qualifications later on (at age 33). The participants in this study were teenagers in the 1970s, and this may, in part, explain these results when, at that time, many families invested less resources in the education of daughters compared to sons. The highest gender correlations were with two personality variables which may explain these results. As in nearly all other studies, the results showed that females were more neurotic and particularly more agreeable than males [62]. The industrial/organizational psychology literature is clear about the personality profile of success at work. It shows that neuroticism is *negatively* correlated with nearly all measures of work success including salary. Furthermore, the extensive literature on sex differences in the Big Five show the same results as found in this study: namely that females are higher than males for those factors negatively associated with success. However, the results in the regression suggests that gender alone, along with various other variables is associated with income. This no doubt could be partly explained by the fact that more women than men had part-time jobs or were unemployed because of family responsibilities.

## 5. Limitations

The present study is based on available variables in the dataset rather than being based on a study designed for the purpose. It would have been desirable to have a second measure of intelligence, say around the age of 20 years to look for changes and whether the correlations would have changed.

More importantly, we had to rely on self-reported income, which could be prone to various errors. It would have been preferable to have a more objective measure such as tax return information as well as dates on things like savings and net wealth.

Another limitation is that personality was assessed at age 50, only four years before income. By this stage of life, individuals tend to be well-established in their careers and relatively fixed in terms of their occupational prestige and income. As income is relatively fixed at this stage there is little room for personality traits at age 50 to impact subsequent income. It is also possible that occupational experiences may reciprocally cause personality change: individuals’ previous occupational environments (and previous income) may have causally influenced their personality traits up until age 50. To some extent, this renders any interpretations of personality as causally influencing the income criterion tentative. There is a considerable debate as to what extent, when and why personality changes over the lifespan [59]. However, systematic mean level changes in personality and individual idiosyncratic patterns of personality change in response to life experiences and events have been well-established: personality traits can and do change throughout adulthood [63,64]. Thus, it would have been most desirable to have robust measures of personality at ages somewhere between 20 and 30 years of age.

## Figures and Tables

**Table 1:** Pearson correlations matrix for the variables examined in the study.

*Variables*		Mean Male SD		Correlation											
			1	2	3	4	5	6	7	8	9	10	11	12	13
1.	Adult earning ability	3.12 (0.42)	_												
2.	Gender	0.53 (0.50)	−0.252 ***	_											
3.	Parental social class	3.28 (1.23)	0.166 ***	−0.015	_										
4.	Childhood cognitive ability	103.8 (12.90)	0.235 ***	0.070 ***	0.260 ***	_									
5.	Educational qualifications	2.67 (1.45)	0.381 ***	−0.114 ***	0.317 ***	0.471 ***	_								
6.	Extraversion	29.33 (6.75)	0.092 ***	0.087 ***	0.020	0.019	0.061 ***	_							
7.	Emotional stability	28.91 (7.00)	0.102 ***	−0.151 ***	−0.001	0.072 ***	0.070 ***	0.208 ***	_						
8.	Agreeableness	37.04 (5.27)	**−0.002**	0.395 ***	0.057 ***	0.106 ***	0.066 ***	0.368 ***	0.045 **	_					
9.	Conscientiousness	34.11 (5.16)	0.080 ***	0.087 ***	−0.006	0.018	0.051 **	0.136 ***	0.164 ***	0.263 ***	_				
10.	Openness	32.43 (5.12)	0.221 ***	−0.030	0.138 ***	0.246 ***	0.312 ***	0.404 ***	0.094 ***	0.322 ***	0.209 ***	_			
11.	Occupational prestige	4.05 (1.23)	0.467 ***	−0.012	0.221 ***	0.359 ***	0.473 ***	0.126 ***	0.073 ***	0.122 ***	0.102 ***	0.255 ***			
12	Married	0.73 (0.44)	0.039 **	−0.069 ***	−0.002	0.035 *	0.039 *	0.021	0.034 *	0.001	0.020	−0.033 *	0.045 **		
13.	Never married	0.9 (0.29)	**0.011**	−0.034 *	0.019	0.004	0.052 **	−0.094 ***	−0.021	−0.082 ***	−0.040 *	0.021	−0.005	_	
14.	Divorced/separated	0.16 (0.36)	−0.034 *	−0.083 ***	0.001	−0.025	−0.070 ***	0.057 **	−0.024	0.064 ***	0.011	0.028	−0.031 *	_	_

*Note:* * *p* < 0.05; ** *p* < 0.01; *** *p* < 0.001. Standard deviations (SD) are given in parentheses. Variables were scored such that a higher score indicated being female (0 = male, 1 = female), a higher score on current earnings, a more professional occupation for parents and cohort members, higher cognitive ability test scores in childhood, highest educational qualification, married, never married, divorced/separated, higher scores on extraversion, emotional stability, agreeableness, conscientiousness, and openness.

**Table 2:** Predicting adult earning ability from childhood and adulthood psychological and socio-demographic variables.

Measures	*β*	*t*	*p*
Gender	−0.240 ***	15.65	<0.001
Parental social class	0.030	1.81	0.071
Childhood cognitive ability	0.046 **	2.91	0.004
Educational qualifications	0.125 ***	7.63	<0.001
*Personality traits*			
Extraversion	0.029	1.83	0.067
Emotional stability	0.004	0.25	0.801
Agreeableness	−0.014	0.84	0.399
Conscientiousness	0.039 **	2.76	0.006
Openness	0.039 **	2.47	0.014
*Marital status* (*being married as the reference group*)			
Never married	0.001	0.11	0.916
Divorced/separated	0.011	0.79	0.432
Occupational prestige	0.371 ***	24.14	<0.001
Variance explained	*R^2^* adjusted = 0.30, *F* (13,4415) = 149.26 ***		

* *p* < 0.05; ** *p* < 0.01; *** *p* < 0.001.

## References

[1] Furnham A., Zeigler-Hill V., Shackelford T.K. (2017). Personality and Occupational Success. The SAGE Handbook of Personality and Individual Differences.

[2] Strenze T. (2007). Intelligence and socioeconomic success. Intelligence.

[3] Su R. (2012). The Power of Vocational Interests and Interest Congruence in Predicting Career Success. Ph.D. Thesis.

[4] Roberts B., Caspi A., Moffitt T. (2003). Work experiences and personality development in young adulthood. J. Personal. Soc. Psychol..

[5] Ng T.W.H., Eby L.T., Sorensen K.L., Feldman D.C. (2005). Predictors of objective and subjective career success: A meta-analysis. Pers. Psychol..

[6] Ng T.W.H., Feldman D.C. (2014). Subjective career success: A meta-analytic review. J. Vocat. Behav..

[7] Seibert S.E., Crant J.M., Kraimer M.L. (1999). Proactive personality and career success. J. Appl. Psychol..

[8] Judge T.A., Cable D.M., Boudreau J.W., Bretz R.D. (1995). An empirical investigation of the predictors of executive career success. Pers. Psychol..

[9] Judge T.A., Higgins C.A., Thoresen C.J., Barrick M.R. (1999). The big five personality traits, general mental ability, and career success across the life span. Pers. Psychol..

[10] Ng T.W.H., Feldman D.C. (2010). Human capital and objective indicators of career success: The mediating effects of cognitive ability and conscientiousness. J. Occup. Organ. Psychol..

[11] Sutin A., Costa P., Miech R., Eaton W. (2009). Personality and career success. Eur. J. Personal..

[12] Hirschi A., Jaensch V.K. (2015). Narcissism and career success: Occupational self-efficacy and career engagement as mediators. Personal. Individ. Differ..

[13] Child D. (1969). A comparative study of personality, intelligence and social class in a technological university. Br. J. Educ. Psychol..

[14] Kiker B., Condon C. (1981). The influence of socioeconomic background on the earnings on young men. Hum. Relat..

[15] Zagorsky J. (2007). Do you have to be smart to be rich?. Intelligence.

[16] Yang B., Lester D. (2016). Personality traits and economic activity. Appl. Econ..

[17] Furnham A., Cheng H. (2017). Socio-demographic indicators, intelligence, and locus of control as predictors of adult financial status. J. Bus. Psychol..

[18] Rode J., Arthaud-Day M., Mooney C., Near J., Baldwin T. (2008). Ability and personality predictors of salary, perceived job success, and perceived career success in the initial career change. Int. J. Sel. Assess..

[19] Wai J., Lincoln D. (2016). Investigating the right tail of wealth. Intelligence.

[20] Abele A., Spurk D. (2009). How do objective and subjective career success interrelate over time?. J. Occup. Organ. Psychol..

[21] Spurk D., Abele A. (2011). Who earns more and why? A multiple mediation model from personality to salary. J. Bus. Psychol..

[22] Le K., Donnellan M., Conger R. (2014). Personality development at work. J. Personal..

[23] Converse P.D., Piccone K.A., Tocci M.C. (2014). Childhood self-control, adolescent behavior, and career success. Personal. Individ. Differ..

[24] Deary I.J., Taylor M.D., Hart C.L., Wilson V., Smith D.G., Blane D., Starr J.M. (2005). Intergenerational mobility and mid-life status attainment: Influences of childhood intelligence, childhood social factors, and education. Intelligence.

[25] Schoon I. (2010). Childhood cognitive ability and adult academic attainment: Evidence from three British cohort studies. Longitud. Life Course Stud..

[26] Spinath B., Spinath F.M., Harlaar N., Plomin R. (2006). Predicting school achievement from general cognitive ability, self-perceived ability, and intrinsic value. Intelligence.

[27] Tong S., Baghurst P., Vimpani G., McMichael A. (2007). Socioeconomic position, maternal IQ, home environment, and cognitive development. J. Paediatr..

[28] Breen R. (2010). Educational Expansion and Social Mobility in the 20th Century. Soc. Forces.

[29] Erikson R., Goldthorpe J.H. (2010). Has social mobility in Britain decreased? Reconciling divergent findings on income and class mobility. Br. J. Sociol..

[30] Furnham A., Argyle M. (1998). The Psychology of Money.

[31] Geyer S., Hemstrom O., Peter R., Vagero D. (2006). Education, income and occupational class cannot be used interchangeably in social epidemiology. J. Epidemiol. Community Health.

[32] Haveman R., Smeeding T. (2006). The role of higher education in social mobility. Future Child..

[33] Heath A. (1981). Social Mobility.

[34] Von Stumm S., Macintyre S., Batty D., Clark H., Deary I. (2010). Intelligence, social class of origin, childhood behaviour disturbance and education as predictors of status attainment in midlife in men. Intelligence.

[35] Harrell T. (1969). The personality of high earning MBA’s: A 20-year longitudinal study. Hum. Perform..

[36] Bowles S.H., Gintis H., Osborne M. (2001). The determinants of earnings. J. Econ. Lit..

[37] Boudreau J., Boswell W., Judge T. (2001). Effects of personality and executive career success in the United States and Europe. J. Vocat. Behav..

[38] Groves M. (2005). How important is your personality? Labor market returns to personality for women in the US and UK. J. Econ. Psychol..

[39] Palifka B. (2009). Personality and income in Mexico. J. Econ. Psychol..

[40] Linz S., Semykina A. (2009). Personality traits as performance enhancers. J. Econ. Psychol..

[41] Judge T. (2009). Core self-evaluations and work success. Curr. Direct. Psychol. Sci..

[42] Judge T., Cable D. (2004). The effect of physical height on workplace success and income. J. Appl. Psychol..

[43] Judge T., Hurst C., Simon L. (2009). Does it pay to be smart, attractive, or confident (or all three)? Relationships among general mental ability, physical attractiveness, core self-evaluations and income. J. Appl. Psychol..

[44] Judge T., Hurst C. (2008). How the rich (and happy) get richer (and happier): Relationship of core self-evaluations to trajectories in attaining work success. J. Appl. Psychol..

[45] Schmidt F., Hunter J. (2004). General mental ability in the world of work. J. Personal. Soc. Psychol..

[46] Nyhus E., Pons E. (2005). The effects of personality on earnings. J. Econ. Psychol..

[47] Gelissen J., de Graaf P. (2006). Personality, social background and occupational career success. Soc. Sci. Res..

[48] Haberfeld Y. (1992). Pay, valence of pay and gender. J. Econ. Psychol..

[49] Judge T., Livingstone B. (2008). Is the gap more than gender? A Longitudinal analysis of gender, gender role orientation, and earnings. J. Appl. Psychol..

[50] Semykina A., Linz S. (2007). Gender differences in personality and earnings. J. Econ. Psychol..

[51] Ferri E., Bynner J., Wadsworth M. (2003). Changing Britain, Changing Lives: Three Generations at the Turn of the Century.

[52] Davie R., Butler N., Goldstein H. (1972). From Birth to Seven.

[53] Fogelman K. (1976). Britain’s 16-Year-Olds.

[54] Marsh C., Burgess R. (1986). Social class and occupation. Key Variables in Social Investigation.

[55] Leete R., Fox J. (1977). Registrar General’s social classes: Origins and users. Popul. Trends.

[56] Douglas J.W.B. (1964). The Home and the School.

[57] Goldberg L.R., Mervielde I., Deary I., De Fruyt F., Ostendorf F. (1999). A broad-bandwidth, public domain, personality inventory measuring the lowerlevel facets of several five-factor models. Personality Psychology in Europe.

[58] Paterson T.A., Harms P.D., Steel P., Crede M. (2016). An assessment of the magnitude of effect sizes. J. Leadersh. Organ. Stud..

[59] Furnham A., Cheng H. (2015). The stability and change of malaise scores over 27 years: Findings from a nationally representative sample. Personal. Individ. Differ..

[60] Dweck C. (2007). Mindset.

[61] Deary I.J., Whalley L.J., Starr J.M. (2009). A Lifetime of Intelligence.

[62] Furnham A. (2008). Personality and Intelligence at Work.

[63] Wille B., Hofmans J., Feys M., De Fruyt F. (2014). Maturation of work attitudes: Correlated change with Big Five personality traits and reciprocal effects over 15 years. J. Organ. Behav..

[64] Roberts B.W., Walton K.E., Viechtbauer W. (2006). Patterns of mean-level change in personality traits across the life course: A meta-analysis of longitudinal studies. Psychol. Bull..

